# Effects of Swimming Exercise on Early Adolescents’ Physical Conditioning and Physical Health: A Systematic Review

**DOI:** 10.3390/jfmk9030158

**Published:** 2024-09-04

**Authors:** Francisco A. Ferreira, Catarina C. Santos, António L. Palmeira, Ricardo J. Fernandes, Mário J. Costa

**Affiliations:** 1 Centre of Research, Education, Innovation and Intervention in Sport (CIFI2D), Faculty of Sport, University of Porto, 4050-450 Porto, Portugal; up202100039@up.pt (F.A.F.); cmsantos@fade.up.pt (C.C.S.); antonio.palmeira@ulusofona.pt (A.L.P.); ricfer@fade.up.pt (R.J.F.); 2 Porto Biomechanics Laboratory (LABIOMEP), Faculty of Sport, University of Porto, 4050-450 Porto, Portugal; 3 Polytechnic Institute of Coimbra, Coimbra Education School, 3030-329 Coimbra, Portugal; 4 Department of Sport Sciences, Higher Institute of Educational Sciences of the Douro (ISCE-Douro), 4560-547 Penafiel, Portugal; 5 Centro de Investigação em Desporto, Educação Física, Exercício e Saúde (CIDEFES), Universidade Lusófona, 1749-024 Lisboa, Portugal

**Keywords:** swimming, longitudinal, physical fitness, sports medicine, youth

## Abstract

Background/Objectives: Swimming is a popular and cost-effective way to prevent sedentary behavior and improve physical conditioning and health during early adolescence. However, information on its impact and benefits on daily life activities is lacking. This systematic review aims to summarize the chronic effects of swimming on physical conditioning and physical health outcomes in early adolescents. Methods: The PRISMA 2020 guidelines were followed and PubMed, Scopus, Web of Science, and International Symposium of Biomechanics and Medicine in Swimming proceedings databases were searched. Eligibility criteria were defined on the PICOS framework (healthy adolescents in early puberty, swimming programmes or training, passive or active control groups, general effects on physical conditioning or health, longitudinal) and risk of bias was assessed using RoBANS 2. Results: From 2365 records, 20 non-randomized studies met the defined criteria. High heterogeneity in sample size and intervention was observed. While studies related to physical conditioning (*n* = 5) focused on physiological variables and muscular function, the evidence regarding physical health outcomes (*n* = 15) explored bone accrual, haemodynamics, body composition, musculoskeletal system, and lung growth. High overall risk of bias (70%) was observed due to strict criteria. Conclusions: Swimming exercise seems to improve cardiorespiratory fitness, cardiac output, haemodynamics, heart growth, motor performance, and body composition of early adolescents. Despite clear evidence that exists on these chronic effects, research on bone health, postural deficit, motor skills, and sleep quality is still missing.

## 1. Introduction

Health is a state of complete physical, mental, and social well-being and not merely the absence of disease or infirmity [1]. From a conceptual point of view, health can be categorized by its physical or conditioning outcomes [2], which are essential for a balanced and fulfilling life [3]. Physical health may include growth, development, somatic experience, and physiological functioning during adolescence (e.g., body size and composition, obesity status, growth trajectories, motor function, and sleep) [2]. In contrast, physical conditioning is a narrower concept that encompasses various fitness aspects like endurance, flexibility, and strength [4].

Engaging in physical activity during puberty is crucial for improving physical health and conditioning throughout life [5]. Similarly, regular exercise (that can be framed within the physical activity concept) has been considered an unstable behavior throughout life, where higher rates of participation in adolescence relate well with its maintenance in adulthood [6,7,8]. Research on this topic has developed evidence mostly on later childhood ages, while early adolescence has been the most neglected period of development. Early adolescence, framed in the age range from 10 to 15 years old [9], is marked by significant physical changes (transitioning from a childlike appearance to a more adult-like physique), cognitive development (including the major shift from primary to secondary education or, in some cases, early school-leaving), and social adjustments (with increasing influence from peer groups beyond the family) [10]. From these risks, preventing sedentary behavior is one of the main strategies of achieving healthy early adolescence [10].

Sedentary behavior is any waking behavior characterized by low energy expenditure (i.e., ≤1.5 metabolic equivalents, such as sitting), which may lead to insufficient physical activity (or physical inactivity as a consequence of not meeting general guidelines) [11]. Recent updates on these independent but well-connected risk factors, i.e., sedentary behavior and physical inactivity, presented negative results worldwide [12] and may explain several issues (like chronic or cardiovascular diseases or mortality [11,13]). Overweight/obesity is, by far, the largest public health problem in early adolescence [14] mediating the motor learning decline in adolescents and children [15], which is closely related to physical inactivity [8,15]. So, encouraging interventions targeting high-risk groups to reduce sedentary behaviors must prevail [6,10].

Regular exercise has been shown to present various benefits in early adolescence, particularly on cognitive function, cardiorespiratory or muscular fitness [16], bone health [17], quality of sleep [18], and mental health (depression, and emotional and behavioural difficulties [19]), as well as on motor skills [20]. Engagement in any regular exercise should be seen like a protective factor for good physical and mental health in children and adolescents, and a non-expensive approach to reduce health issues [11]. General guidelines recommend at least 60 min of moderate- to vigorous-intensity (mostly aerobic) daily regular exercise for children and adolescents, including aerobic, muscle- and bone-strengthening activity [20].

From the various exercise modes, swimming has become one of the most regular at early ages and is a suitable option for meeting guidelines [20]. The initial practice in swimming is related to safety due to the high drowning episodes in children [21]. This choice is also related to multiple health benefits known, mainly in physiological parameters [22,23,24]. Additionally, swimming induces improvement in cardiorespiratory fitness and overall cardiovascular risk in overweight and obese children [25]. However, regular and systematic swimming training seems to impair sleep quality/quantity [26] and bone health [27].

Despite literature presenting information on how swimming helps develop the various aspects of motor skills in children [28] or biological indices in adulthood [29], there seems to be a lack of systematic evidence related to the early adolescence period. A review examining the chronic effects of swimming exercise on different parameters will allow understanding of the true impact on this age group in daily life. This systematic review aims to summarize the available literature on the chronic effects of swimming exercise on physical conditioning and physical health outcomes in healthy early adolescents. It will provide a synthesis for the different parameters assessed, describe the different methods used, and draw implications on adolescents aged 10–15 years for future research.

## 2. Materials and Methods

This systematic review was designed in accordance with the Preferred Reporting Items for Systematic Reviews and Meta-Analyses (PRISMA) 2020 statement [30]. The review methodology and protocol registration preceded the search. The protocol for this study was registered on the INPLASY database (registration number INPLASY2023100078), which was published on 23 October 2023 and is available in full on: https://inplasy.com/inplasy-2023-10-0078/ (accessed on 23 October 2023) [31].

### 2.1. Eligibility Criteria

Peer-reviewed articles searching the current review scope were eligible, without limitation on language (as long as the studies comprised the title and abstract written in English) or publication date, and no filtering application to increase the chances of identifying appropriate studies. Non-peer-reviewed articles/journals, reviews (i.e., qualitative review, systematic review, meta-analysis), books, book chapters, commentaries, editorials, letters to the editor, overviews, dissertations, theses, or trial registrations were excluded from the analysis.

The initial search was conducted up to 18 December 2023 using three electronic databases (PubMed, Scopus, and Web of Science) as well as the International Symposium of Biomechanics and Medicine in Swimming (BMS) proceedings (from 1970 to 2023) due to its relevance in swimming-specific context (available online: https://www.iat.uni-leipzig.de/datenbanken/iks/bms/ (accessed on 18 December 2023) [32]). After data extraction, additional records were retrieved using manual search, snowballing citation tracking (references and cited by) and expert consultation (knowledge and research in swimming). The eligibility criteria were defined according to the PICOS (population, intervention, comparison, outcome, and study design) framework (presented in Table 1). The records had to include participants between 10 and 15 years of age to be considered valid, even if the reported mean age was below 10.00 or above 15.99 years.

### 2.2. Search Strategy

The Boolean search method (including AND/OR) was used to search literature covering terms related to physical conditioning variables and physical health outcomes. In the three databases (PubMed, Scopus, and Web of Science), the terms had to be presented in the title, abstract, or keywords (using the search by “title/abstract”, “title-abstract-key”, and “topic”, respectively). Meanwhile, the terms in the International Symposium of Biomechanics and Medicine in Swimming proceedings had to be presented in all fields. Search lines selected contained: (1) (“swim*”); AND (2) (“adolescen*” OR “pedriatri*” OR “teenag*” OR “youth” OR “young*” OR “age group*” OR “(pre)pubert*”); AND (3) (“intervent*” OR “program*” OR “train*” OR “lesson*” OR “exercise*”); AND (4) (“health*” OR “physical condition*” OR “fitness”).

### 2.3. Selection Process and Data Extraction

After extraction, the records retrieved from databases were screened independently by two authors (FAF and CCS), and automated duplicates removal was performed using EndNote 20.6 for Windows (ClarivateTM, Philadelphia, PA, USA). Firstly, all data was analyzed by titles and abstracts and then with full-text selection. Records were extracted into a tailored Microsoft^®^ Excel 2016 worksheet (Microsoft Corporation, Redmond, WA, USA) created for data summary.

FAF and CCS performed a completed and independent data extraction to group the following physical conditioning or physical health outcomes. In case of disagreements, a third author (MJC) provided arbitrage and was allowed to reach a consensus. Information was synthesized by: (i) author(s) and year of publication; (ii) country; (iii) sample characteristics (like group, sample size, sex, age); (iv) intervention characteristics (e.g., context, duration, frequency, session duration, or distance); (v) assessments (assessed domains, aim, and variables); (vi) main results.

### 2.4. Study Risk of Bias

An independent reviewer (FAF) performed the risk-of-bias analysis of each included record, and disagreements were solved by another author (MJC). The Revised Risk of Bias Assessment Tool for Nonrandomized Studies of Interventions (RoBANS 2 [33]) was used for the risk-of-bias assessment. The tool includes eight domains (comparability of the target group, target group selection, confounders, measurement of intervention/exposure, blinding of assessors, outcome assessment, incomplete outcome data, and selective outcome reporting) assessed with one of three judgments (low, unclear, or high). Since RoBANS 2 allows different approaches for assessing overall bias [33], the selected method was to use the worst risk of bias identified among three domains (confounders, measurement of intervention/exposure, and incomplete outcome data) due to its high relevance for the current scope.

## 3. Results

### 3.1. Study Selection

**Figure 1:**
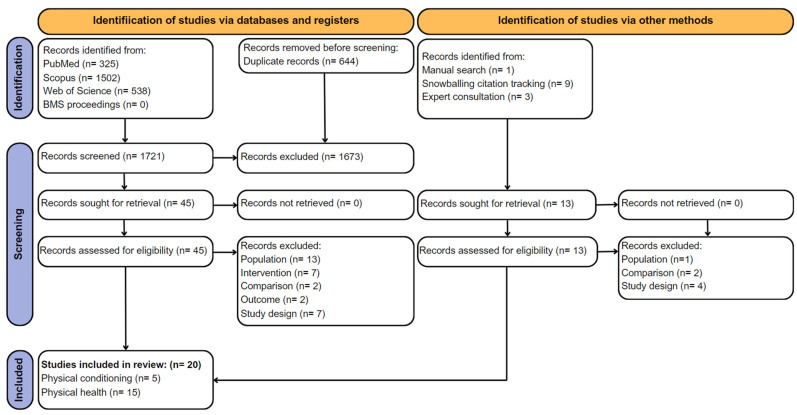
PRISMA 2020 flow chart for studies’ identification, screening, and inclusion.

A total of 2365 potentially relevant records were identified from PubMed (*n* = 325), Scopus (*n* = 1502), and Web of Science (*n* = 538). From those, 644 duplicates were then excluded, leaving 1721 references to be screened by title and 45 remaining sought for retrieval (removed *n* = 1673). Also, 31 records were removed due to the PICOS framework’s exclusion criteria (P: *n* = 13, I: *n* = 7, C: *n* = 2, O: *n* = 2, S: *n* = 7). Additional records (*n* = 13) were identified from other methods (manual search, snowballing citation tracking, and expert consultation), and some (*n* = 7) were excluded (P: *n* = 1, C: *n* = 2, S: *n* = 4). At the end, 20 relevant studies were identified and included in the present review for further analysis and divided into physical conditioning (*n* = 4 from databases and *n* = 1 from other sources) or physical health (*n* = 10 from databases and *n* = 5 from other sources) outcomes. Figure 1 shows the flow chart of the detailed process for the studies’ selection.

### 3.2. Study Characteristics

According to the full search, 20 articles were included in the final analysis. All studies presented a non-randomized controlled design, and their characteristics were divided and categorized by physical conditioning (*n* = 5, [34,35,36,37,38]) or physical health (*n* = 15, [39,40,41,42,43,44,45,46,47,48,49,50,51,52,53]). The publications dated between 1979–2022 and interventions were performed in 12 countries (United Kingdom, *n* = 5; Poland, *n* = 3; Portugal, *n* = 2; Brazil, Canada, China, France, Germany, Italy, Russia, Spain, and United States of America, *n* = 1). Apart from the swimming group, 10 studies (50%) included only a passive control and/or placebo group [34,35,37,38,39,40,42,43,46,49], while the other half (50%) included at least one active control group regarding other sports [36,41,44,45,47,48,50,51,52,53].

Despite the observed sample size variability (between 21–234 participants), most studies (80%) included 40 or more participants [36,37,38,39,40,41,42,43,44,45,47,48,49,50,52,53] and the remainder included at least 20 participants [34,35,46,51]. Ten studies (50%) included both sexes [34,35,36,37,39,40,44,49,50,52], eight studies (40%) included only males [38,42,43,45,47,48,51,53], and two studies (10%) included only females [41,46]. The lowest age found was 9.68 ± 0.71 years [34], and the highest was 16.30 ± 1.20 years [41]. Regarding the swimming group, 90% of the studies (*n* = 18) reported participation with the context of training [34,35,36,37,39,41,42,43,44,45,46,47,48,49,50,51,52,53], while the others 10% (*n* = 2) participated in recreational swimming [38,40].

In line with intervention procedures applied to the experimental groups (i.e., swimming or other sports groups), a large heterogeneity was observed among the research design. One of the main differences was the period chosen for the follow-up ranging from 3 to 36 months ([35,52] and [38], respectively). Still, 11 (55%) studies presented a period equal to or higher than 12 months [35,36,38,40,45,47,48,49,50,51,53].

### 3.3. Main Findings Regarding Physical Conditioning Outcomes

The studies included in the physical conditioning outcomes are presented in Table 2. During the follow-up period, the participants were evaluated two [34,36], three [35,37], or six times [38]. The variables assessed were mainly related to muscular function (strength and resistance [34,37]) and physiological parameters (cardiorespiratory fitness, heart rate, and other related [35,36,38]) and were also linked to body-composition traits (like anthropometrics, maturity, body segments length, breadth or girth [35,37,38]).

Swimming for 6 or 7 months was beneficial for muscular endurance but did not improve muscular strength [34,37]. Even 36 months of recreational swimming was enough to improve cardiorespiratory fitness [38], and this was independent of maturational status [35,37] or sport-specific performance [36]. The swimming training also promotes morphological and functional adaptations, resulting in higher cardiac output and stroke volume [35].

### 3.4. Main Findings Regarding Physical Health Outcomes

The studies included in the physical health domain are presented in Table 3. The number of follow-up assessments comprised two [39,41,42,43,44,45,46,47,48,52,53], three [40,49,50], and five [51] testing moments. The most observed variables evaluated were related to the development and composition of bones [41,44,45,46,47,48,49,50,53]. The remaining studies focused on diversified aims of hemodynamic and heart rate variability [39], postural defect occurrence [40], shoulder rotator-cuff balance [42], biatrial remodelling [44], lung growth [46], physiological capacity or motor abilities related with anthropometric measures [51], and body composition [52].

The studies regarding the effects of swimming on bone density presented mixed findings. While some studies found no effects of swimming on changing bone properties [41,44,47,49], others observed bone mineral content, structure, and mass improvements but in lower magnitude when compared to other sports (mainly, the considered osteogenic sports [45,48,53]). Swimming seems to positively affect the regulation and stability of the circulatory system, the vagal heart rate variability, and the index of vagosympathetic interaction [39]. Improvements were also observed in heart growth (presenting morphological adaptations [43]) and in physiological and motor performance [51]. Positive changes were also registered in anthropometric measures and body composition [51,52]. One study observed that swimming did not lead to changes in postural deficits (i.e., the frequently concurrent scoliotic posture and/or shoulder asymmetry [40]), while other evidenced an increased muscular imbalance in the shoulder rotators [42]. Swimming also did not seem to promote lung growth of practitioners [46].

### 3.5. Risk of Bias

**Figure 2:**
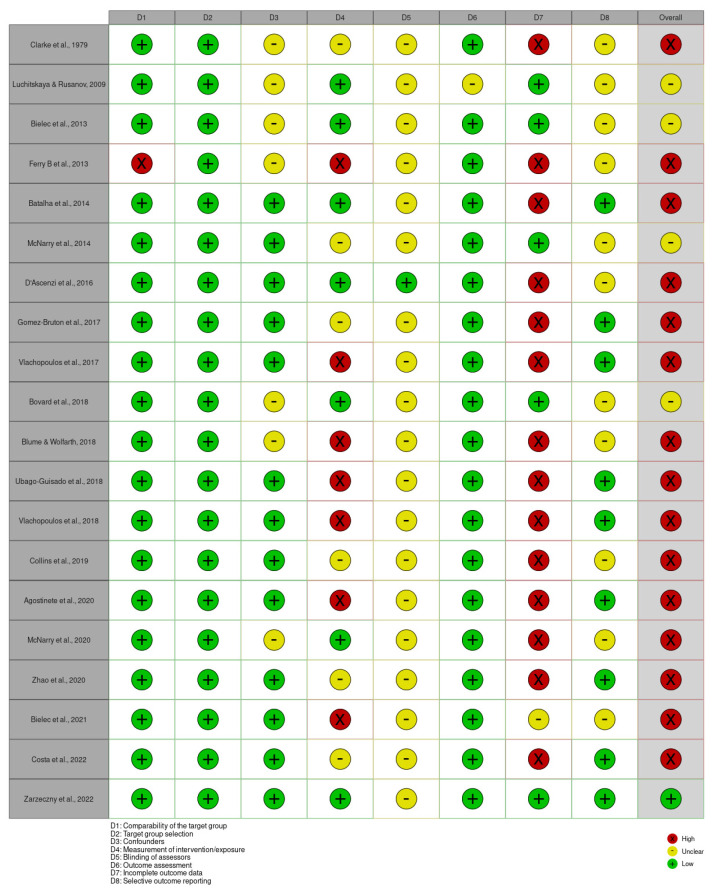
Low (+), unclear (−), or high (×) judgments by domain and overall bias for each study [34,35,36,37,38,39,40,41,42,43,44,45,46,47,48,49,50,51,52,53] (robvis tool [54]).

Figure 2 presents the risk of bias judgement using the RoBANS 2 tool. None of the studies was classified as low in all domains. There were five studies that did not present any high judgment [35,38,39,40,46] and were classified as unclear [35,39,40,46] or low [38] in overall bias. The remaining studies presented high risk of bias.

**Figure 3:**
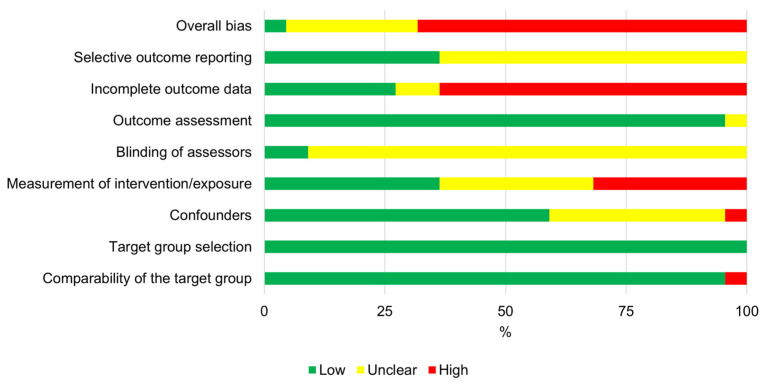
Risk-of-bias domains and overall bias are presented as percentages across the included studies.

Relative judgments (% of low, unclear, or high) observed for each domain and an overall bias related to all studies assessed are presented in Figure 3. Incomplete outcome data was the domain with the worst relative risk of bias (70% of high judgment) followed by measurement of intervention/exposure (35% of high judgment), which often presented duration without evaluating inferential statistical [53]. Despite one study presenting one active control group, the lack of a passive control group is presented as a limitation [51]. However, a large observation for the unclear score was presented in the confounders (35%), blinding of assessors (95%), and selective outcome reporting presented (55%) domains. Related to the blinding domain, only one study presented an experienced cardiologist, blinded to the study [43].

The domains with the best values (that means high values of low risk of bias) were the target group selection (100%), comparability of the target group (95%), outcome assessment (95%), and confounders (60%). The reason for the lowest bias in the target group selection among all domains is due to prospective study designs [36]. The main confounding variables presented were the maturation status [44,45] and even the exposure to resistance training [50]. Overall bias presented 75% for high-risk judgment, followed by 20% for unclear.

## 4. Discussion

The purpose of the current investigation was to review the evidence regarding the chronic effects of swimming exercise on physical conditioning and physical health outcomes in healthy early adolescents. The focus was the comparison of the swimming group with active (other sports) or passive (nonathletes) control groups, and their effects on multiple variables of physical conditioning and health were evaluated. There are some longitudinal studies conducted in swimming, but it is still difficult to draw clear conclusions. The main findings highlight that swimming exercise improves cardiorespiratory fitness, cardiac output, haemodynamic function, heart growth, motor performance, anthropometry, and body composition in early adolescents. Despite the results being divided into physical conditioning and health outcomes, in some cases, it was difficult to split due to the strict connection between both [51], and the main outcomes were allocated to the preferential characteristics.

Overall bias was developed and assessed according to three main domains chosen due to their importance to the current aim. Since the age range is wide (between 10 to 15 years), which often suggests heterogeneity in the maturational status [9], the confounders domain was selected to mitigate the distortion or bias of the results between the variables under investigation (results did not change when maturity was used as a confounder [45]). Given that studies should require an intervention period to check the behavior of certain variables among the swimming group across time, the measurement of the intervention/exposure domain presented high relevance. Due to the comparison of swimming and active or passive control groups was necessary, the incomplete outcome data domain regarding baseline differences between groups was the third domain selected to reflect the overall bias.

Although some domains were well-rated, poor results of the previous three domains exposed (mainly measurement of intervention/exposure and incomplete outcome data domains) reflect a general high risk in overall bias. The main reasons are related to the absence of confounding variables (such as maturity), which sometimes require sensitive procedures to be implemented and then are discarded from the analysis. The groups recruitment, like the baseline existent differences between experimental and controls, and the difficulty in standardizing the swimming and other sports interventions are other problems that researchers deal with and explain the high risk of bias. The 43-year time window between the oldest study and the last one identified may also affect the results as uncontrolled or missing issues were more frequent in the oldest interventions. The three selected domains imply a great deal of rigour in theoverall bias rating, which may allow a reasonable consideration for this concerned risk of bias. Additionally, the longitudinal study designs constituting the scope can be linked and justify the previous outcome [55]. Nevertheless, physical health seems to present a general worst-case scenario when compared to physical conditioning outcomes linked to incomplete outcome data.

### 4.1. Main Findings on Physical Conditioning

The main results related to muscular strength presented consistency as both 6 [37] and 7 [34] months of swimming did not induce improvements assessed with different methods (handgrip strength and weightlifting, respectively). The narrow association between in-water force production (through tethered swimming [56]) and both upper and lower limbs’ muscular strength [57] enhances clear transfer between environments (dryland and aquatic). However, the dryland methods to assess swimmers’ strength should be specific of swimming movement and its muscular stimulus. In contrast, swimming practice promotes muscular endurance [34], justified by the traditional swimming training approach that has been designed to develop endurance capacity [58,59].

Cardiorespiratory fitness should be assessed when implementing interventions in early adolescents, as lower levels are known to be associated with obesity in this age group [14]. This physiological fitness component is associated with cognitive function, self-worth, and life satisfaction in early adolescents [16], and can also be considered a health-related physical fitness indicator [60]. Maximal cardiorespiratory fitness refers to aerobic power and is defined as the highest rate of oxygen uptake that can be achieved during maximal or exhaustive exercise (VO_2_max [61]). One study demonstrated improvements in cardiorespiratory fitness in swimmers when compared with control subjects [38], corroborating previous literature of significant and meaningful improvements in cardiorespiratory fitness through swimming in healthy adolescents [23]. These results are consistent with the benefits on aerobic response to exercise when swimming comes in as an example [20,22].

Notwithstanding, high response on aerobic performance in swimming seems similar when compared to soccer players, cyclists, and cross-country skiers [36], or even comparable to rowers, runners, and cyclists [62]. It is stated that aerobic response is not affected by maturational status [35,37], but it seems that maximal oxygen uptake is strongly associated with age despite presenting sex-related differences [63]. Conversely, oxygen transportation is increased through changes in haemoglobin concentration, promoted by aerobic swimming [64] as suggested by the current findings [51].

The increasing of cardiac output leading to higher peak oxygen uptake derived from aerobic exercise is described elsewhere [65,66]. Previous studies did not present higher heart rate response to exercise [65], suggesting that cardiac output performance (given by the product of heart rate and stroke volume [67]) is a consequence of stroke volume as presented through the current findings [35]. Furthermore, aerobic power performance in response to higher stroke volumes is linked with higher ventricular dimensions [66,68]. Nevertheless, the responses to exercise differ along the lifespan [69], and stringent evidence appears to be required in ages 10–15 years.

Despite the fact that cross-sectional literature of swimming’s acute effects on physical conditioning outcomes seems wide (mainly in physiology [23]), it is still necessary to clarify the chronic effects on larger samples [22,51].

### 4.2. Main Findings on Physical Health

To normalize haemodynamic stress during exercise, the heart suffers chronic morphological adaptation through mass increase in early adolescents [70], allowing positive effects on cardiovascular health [13]. Likewise, swimming seems to promote those adaptations through the augmented biatrial size [43] with influence on the circulatory system (i.e., cardiovascular [39]). Despite that, chronic effects on right ventricular performance and atrial function seem to be supported by endurance sport-specific adaptation, with lower response in swimming compared to running [71]. The findings suggesting improvements in the index of vagosympathetic interaction (i.e., both sympathetic and parasympathetic nerves) and heart rate variability [39] were expected due to their connection [72], benefiting cardiovascular health [73] mediated by adipose tissue decrease [74].

Physical activity (based on aerobic exercise) promotes the decreasing adiposity and limiting weight gain [75]. In this view, the positive effects of swimming (as a mode of aerobic physical activity) on anthropometric [51] and body composition seem consensual, producing improvements in lean soft tissue [53] and preventing adipose tissue growth [52,53]. Recently, it has been described that there is a narrow association of motor competence with body composition [8,76] and with physical activity through early adolescence [77,78]. In sequence, team sports interventions improve motor performance [79], while swimmers and racket-sports players also present progress without sport-related differences [51].

It was described that bone accrual is determined by the mode of exercise [17] and its context [45,48,53]. Swimming seems not to be beneficial for bone changes [41,44,47,49] but does not comprise negatively accrual [80] and does not present sex-related differences in bone density [27]. Despite that, results regarding its development and health are still fragile and unclear, because some studies are pointing in the direction of low improvements in bone acquisition, mineral content, and density by swimming [45,48,53]. However, swimmers may benefit from adding dryland exercises to their training, as these exercises are more effective than swimming alone for improving bone density [80]. Shoulder asymmetry is closely related to the anatomical condition and one of the first symptoms of scoliotic posture [81]. National-level swimmers present shoulder asymmetries, suggesting that swimming training promotes shoulder imbalances [42]. However, recreational swimmers did not present changes in scoliotic posture and/or shoulder asymmetry [40], suggesting that the stimulus is not enough to considered beneficial or harmful to correct postural deficits. Nevertheless, it is not clear if the exercise background and athletic abilities (i.e., sport participant classification like recreational or competitive) should be considered in terms of swimming effects on shoulder and posture.

Despite lung growth and its relationship with exercise being a thoroughly explored topic [82], recent studies suggest that higher volumes are inherent traits and not a consequence of exposure to swimming and/or other sports [46]. Until the beginning of adulthood, lung development seems to present sex-related differences, with girls being favoured in growth rate [83]. Despite this, the single study with lung growth analysis only included female early adolescents where this age gap is sensitive in lung development [46].

### 4.3. Limitations and Future Research

A spread of up to 10 sessions per week and duration or distance per session diverged, leading to an overall spread in the number of sessions (when information was available). This heterogeneity between and within (when more than one experimental group) studies can present a weakness, and longer follow-ups are needed to allow a true comprehension of swimming’s effects on physical health and its actual relationship with other variables. Due to its concept, which can have far-reaching outcomes, there are many variables yet to be explored. Future research should explore the effects of swimming exercise from a physical health standpoint, including domains such as sleep (quantity and quality) and motor function, and understand how those adaptations may influence daily life activities. Moreover, identification of the most appropriate dose response in terms of volume and intensity that should characterize swimming sessions is also needed. Likewise, new research lines should incorporate ecological approaches using multivariate analyses to capture complex interactions and relationships.

## 5. Conclusions

The effects of swimming exercise during early adolescence are not so well-documented, totaling just 20 papers in the literature. Despite most of the interventions having made a great effort to include passive or active control groups, a high overall bias was observed. The existent studies are primarily limited to physiological variables and morphological health linked to the musculoskeletal system. So, some questions still remain on the reliability of meaningful health indicators for this age group, suggesting a likely ceiling effect for health-related parameters on longitudinal designs.

## Figures and Tables

**Table 1:** Inclusion and exclusion criteria according to PICOS framework.

Framework	Inclusion Criteria	Exclusion Criteria
Population (P)	Healthy adolescents aged between 10–15 years old (inclusive)	All spectrum of disabled adolescents; Practitioners from other aquatic activities (i.e., triathlon, water polo, artistic swimming, water aerobics)
Intervention (I)	Swimming interventions or training programmes	Combined interventions (e.g., swimming and other physical activities or supplementation), where the effects of swimming could not be isolated
Comparison (C)	Passive control groups and/or placebo (not exposed to other interventions and just keep the physical education classes) or active control groups regarding other sports	Comparison between different methods of regular swimming or additional dry-land methods aiming performance
Outcome (O)	Published studies that measured the effects on general physical conditioning and physical health	Studies aiming for performance outcome during sport-specific exercise (e.g., maximal oxygen uptake, stroke rate, speed)
Study design (SD)	Randomized and non-randomized longitudinal designs	Cross-sectional designs

**Table 2:** Included studies regarding the effects of swimming exercise on physical conditioning with respective data synthesis.

Author, Year (Country)	Sample Characteristics (Baseline)		Intervention *		Assessed Domains	Main Findings
	Group (Context)	Sex *n =* Age (Years)	Period (Months)	Weekly Frequency (Per Session)		
Clarke & Vaccaro, 1979 [34] (United States of America)	Swimming (Training)	♂♀ *n* = 15 9.68 ± 0.71	7	four sessions (3.7–9.1 km)	Anthropometry Muscular Strength Muscular endurance	Swimming: ↑ Muscular endurance → Muscular strength → Body composition
	Control (Nonathletes)	♂♀ *n =* 15 10.28 ± 0.55		-------		
McNarry et al., 2014 [35] (United Kingdom)	Swimming (Training)	♂♀ *n* = 19 10.4 ± 1.1	36	NA (6 ± 3 h)	Anthropometry Maturation Training status	Swimming: ↑ VO_2_ peak due to ↑ Q
	Control (Nonathletes)	♂♀ *n* = 15 9.8 ± 0.9		-------		
Blume & Wolfarth, 2018 [36] (Germany)	Swimming (Training)	♂♀ *n* = 18 ♂ 14.7 ± 1.6 ♀ 15.1 ± 1.0	12	♂ 14.2 ± 8.1 h (NA) ♀ 17.0 ± 4.5 h (NA)	Performance	Sport-specific skills: Do not express aerobic performance
	Cross-Country Skiing (Training)	♂♀ *n* = 35 ♂ 15.7 ± 1.2 ♀ 16.1 ± 1.4		♂ 10.6 ± 3.7 h (NA) ♀ 11.7 ± 3.7 h (NA)		
	Soccer (Training)	♀ *n* = 45 13.8 ± 1.4		9.6 ± 2.8 h (NA)		
	Cyclist (Training)	♂♀ *n* = 48 ♂ 13.6 ± 2.0 ♀15.2 ± 1.4		♂ 12.2 ± 3.8 h (NA) ♀ 14.2 ± 4.5 h (NA)		
McNarry et al., 2020 [37] (United Kingdom)	Swimming (Training)	♂♀ *n* = 28 G1: 11.3 ± 1.6 G2: 14.8 ± 1.6	6	G1: NA (10.5 ± 3.3 h) G2: NA (16.4 ± 1.4 h)	Maturation Aerobic Strength Performance	Swimming: No aerobic or strength maturational thresholds presented
	Control (Nonathletes)	♂♀ *n* = 26 G1: 9.7 ± 1.5 G2: 14.4 ± 0.5		-------		
Zarzeczny et al., 2022 [38] (Poland)	Swimming (Recreational)	♀ *n* = 20 10.47 ± 0.30	36	two sessions (45 min)	CRF Anthropometry	Swimming: ↑ CRF
	Control (Nonathletes)	♀ *n* = 20 10.52 ± 0.31		-------		

* related to the swimming or other sport active groups. Abbreviations: ♂—male; ♀—female; ↑—increased; →—not changed; CRF—cardiorespiratory fitness; G1—pre pubertal; G2—pubertal; Q—cardiac output; NA—not available; VO_2_—oxygen uptake.

**Table 3:** Included studies regarding the effects of swimming exercise on physical health with respective data synthesis.

Author, Year (Country)	Sample Characteristics (Baseline)		Intervention *		Assessed Domains	Main Findings
	Group (Context)	Sex *n =* Age (Years)	Period (Months)	Weekly Frequency (Per Session)		
Luchitskaya & Rusanov, 2009 [39] (Russia)	Swimming (Training)	♂♀ *n* = 42 15–16	8	six sessions (2.5 ± 0.5 h)	Central and cerebral haemodynamics Heart rate variability	Swimming: ↑ Regulation of blood circulation ↑ Stability of circulatory system ↑ Heart rate variability
	Control (Nonathletes)	♂♀ *n* = 96 15–16		-------		
Bielec et al., 2013 [40] (Poland)	Swimming (Recreational)	♂♀ *n* = 116 13.4 ± 0.3	24	one session (45 min)	Anthropometry Postural deficit occurrence	Swimming: → Anthropometry → Scapula asymetry
	Control (Nonathletes)	♂♀ *n* = 114 13.4 ± 0.3		-------		
Ferry et al., 2013 [41] (France)	Swimming (Training)	♀ *n* = 26 15.9 ± 1.9	8	five sessions (120 min)	Hip structural BMC BMD	Swimming: → Bone health
	Soccer (Training)	♀ *n* = 15 16.2 ± 0.7		five sessions (120 min)		
	Control (Nonathletes)	♀ *n* = 32 16.3 ± 1.2		-------		
Batalha et al., 2014 [42] (Portugal)	Swimming (Training)	♂ *n* = 27 14.48 ± 0.50	4	6.75 ± 0.86 sessions (126 ± 26.39 min)	Shoulder rotator–cuff balance	Swimming: ↑ Muscular imbalances in the shoulder rotator-cuffs
	Control (Nonathletes)	♂ *n* = 33 14.64 ± 0.49		-------		
D’Ascenzi et al., 2016 [43] (Italy)	Swimming (Training)	♂ *n* = 57 10.8 ± 0.2	5	five to six sessions (75–90 min)	Biatrial remodelling (atrial measurements and myocardial function)	Swimming: Promotes heart growth and morphological adaptations
	Control (Nonathletes)	♂ *n* = 37 10.2 ± 0.2		-------		
Gomez-Bruton et al., 2017 [44] (Spain)	Swimming (Training)	♂♀ *n* = 23 15.0 ± 2.2	8	6 h (NA)	Aereal BMD Bone strength Bone structure	Swimming: → Radius bone strength ↑ Low in tibia bone strength ↑ Low in trcohanter aereal BMD Swim + Weightlifting: ↑ Bone strength
	Swim + Weightlifting (Training)	♂♀ *n* = 11 15.1 ± 2.8		6 h (NA)		
	Control (Nonathletes)	♂♀ *n* = 28 14.1 ± 2.3		-------		
Vlachopoulos et al., 2017 [45] (United Kingdom)	Swimming (Training)	♂ *n* = 37 13.5 ± 1.0	12	9.4 ± 5.1 sessions (NA)	Bone acquisition	Soccer benefits compared with other sports: ↑ Femoral neck and lumbar spine bone acquisition
	Soccer (Training)	♂ *n* = 37 12.9 ± 0.9		10.0 ± 2.3 sessions (NA)		
	Cyclism (Training)	♂ *n* = 28 13.2 ± 1.0		5.2 ± 2.1 sessions (NA)		
	Control (Nonathletes)	♂ *n* = 14 12.3 ± 0.5		-------		
Bovard et al., 2018 [46] (Canada)	Swimming (Training)	♀ *n* = 11 12.4 ± 0.8	7.6	five to seven sessions (NA)	Lung growth	Swimming: → Lung growth (ihnerent characteristic)
	Control (Nonathletes)	♀ *n* = 10 13.2 ± 1.3		-------		
Ubago-Guisado et al., 2018 [47] (United Kingdom)	Swimming (Training)	♂ *n* = 39 13.5 ± 1.0	12	9.5 ± 5.0 sessions (NA)	Aereal BMD Hip geometry Trabecular bone score	Swimming: → Aereal BMD → Hip geometry → Trabecular bone score
	Soccer (Training)	♂ *n* = 37 12.9 ± 0.9		10.0 ± 2.3 sessions (NA)		
	Cyclism (Training)	♂ *n* = 28 13.3 ± 1.1		5.2 ± 2.1 sessions (NA)		
Vlachopoulos et al., 2018 [48] (United Kingdom)	Swimming (Training)	♂ *n* = 37 13.5 ± 1.0	12	9.4 ± 5.1 sessions (NA)	Bone development	Soccer higher compared with other sports: ↑ BMC and bone stiffness
	Soccer (Training)	♂ *n* = 37 12.9 ± 0.9		10.0 ± 2.3 sessions (NA)		
	Cyclism (Training)	♂ *n* = 28 13.2 ± 1.0		5.2 ± 2.1 sessions (NA)		
	Control (Nonathletes)	♂ *n* = 14 12.3 ± 0.5		-------		
Collins et al., 2019 [49] (United States of America)	Swimming (Training)	♂♀ *n* = 128 12.8 ± 2.9	12	5.7 h (NA)	Bone accrual	Swimming: → Total body bone mass and accrual → Hip bone mass and accrual
	Control (Nonathletes)	♂♀ *n* = 106 13.8 ± 3.2		-------		
Agostinete et al., 2020 [50] (Brazil)	Swimming (Training)	♂♀ *n* = 18 12.7 ± 1.2	18	1001 ± 196 min (NA)	Bone accrual	Impact sports higher compared with other sports: ↑ Aereal BMD ↑ Bone mineral apparent density
	Impact (Training)	♂♀ *n* = 33 12.7 ± 1.3		664 ± 369 min (NA)		
	Control (Nonathletes)	♂♀ *n* = 20 12.6 ± 2.6		-------		
Zhao et al., 2020 [51] (China)	Swimming (Training)	♂ *n*= 10 11–13	24	six sessions (2–3 h)	Physiological performance Body dimensions Motor abilities	Both groups develop similar: ↑ Vital capacity ↑ Hemoglobin concentration
	Racket-sports (Training)	♂ *n*= 11 11–13		20.8 h (2–3 h)		
Bielec et al., 2021 [52] (Poland)	Swimming (Training)	♂♀ *n* = 46 11.7	3	8.2 ± 1.4 sessions (NA)	Body composition	Swimming: ↓ Adipose tissue growth
	Other sports (Training)	♂♀ *n* = 42 11.9		3.5 ± 0.9 sessions (NA)		
Costa et al., 2022 [53] (Portugal)	Swimming (Training)	♂ *n* = 20 12.7 ± 0.4	12	NA (NA)	Body composition Bone tissue	Swimming: ↑ Lean soft tissue → Fat mass increments ↑ Aereal BMD (less than soccer)
	Soccer (Training)	♂ *n*= 20 12.4 ± 0.3		NA (NA)		

* Related to the swimming or other sport active groups. Abbreviations: ♂—male; ♀—female; ↑—increased; ↓—decreased; →—not changed; BMC—bone mineral content; BMD—bone mineral density; NA—not available.
